# The Cartesian Gaussian additive noise model for directed network inference in omics data

**DOI:** 10.1186/s12874-026-02849-6

**Published:** 2026-04-22

**Authors:** Bailey Andrew, David R. Westhead, Luisa Cutillo

**Affiliations:** 1 School of Computer Science, University of Leeds, Leeds, LS2 9JT UK; 2 School of Molecular and Cellular Biology, University of Leeds, Leeds, LS2 9JT UK; 3 School of Mathematics, University of Leeds, Leeds, LS2 9JT UK

**Keywords:** Causal inference, Graphical models, Kronecker-structured models, Covariance estimation

## Abstract

**Background:**

Access to omics datasets, such as single-cell RNA-sequencing, enables us to estimate the regulatory networks governing the differentiation, proliferation, and interaction of cells in our body. Knowledge of such networks can give us valuable insight on the structure and progression of diseases; the difficulty is in estimating them. Most methods that estimate these networks either make an independence assumption (‘the cells in your body do not interact’), or ignore the directional nature of gene regulation.

**Methodology:**

In this paper, we introduce the Cartesian Linear Gaussian Additive Noise Model to learn both cell-cell and gene-gene interactions. Our method is a statistical method; it is fit with maximum likelihood estimation, and we prove that a unique optimum always exists (under certain assumptions) using tools from high-dimensional statistics.

**Results:**

Our method differs from prior work in its lack of an independence assumption; we show that this leads to a real improvement in gene regulatory network and cell network reconstructions relative to analogous independence-assuming methods.

**Conclusions:**

We have developed and proved viable a novel method that learns directed gene regulatory networks, without assuming independence of cells. Our method is also extensible to more complicated omics datasets, such as longitudinal bulk RNA-sequencing datasets, through its ability to handle ‘tensor-variate’ datasets.

**Trial Registration:**

Clinical trial number: not applicable.

## Introduction

Knowledge of gene regulatory networks (GRNs) can give insights into potential therapeutic targets [1], making them of keen interest to the fields of biology and medicine. There are many methods that estimate gene correlation networks (such as WCGNA [2]), but such methods learn undirected networks that cannot not capture the directed nature of gene interactions. Others, such as ARACNE [3], are able to learn directed networks, but do not take into account that the cells come from a heterogeneous, interacting environment (i.e. it makes an *independence assumption*). This assumption weakens the utility of the learned network.

Recently, multi-axis methods like scBiGLasso [4] have been developed to account for this. Such methods, often called ‘Kronecker-structured’ methods due to their use of the Kronecker product, have the added benefit of *simultaneously learning a network of cells*. By analyzing such networks, the same tool can be used for cell subtyping and gene regulatory inference, and both networks benefit from knowledge of the other. However, none of these methods learn directed graphs.

In this paper, we present a method to simultaneously learn a directed gene and cell network from scRNA-seq data. In fact, our methodology can be applied to any matrix-variate dataset, such as other omics data, and even any ‘tensor-variate dataset’ (datasets that need three or more indices to fully describe, such as longitudinal bulk omics data). Our method enables us to extract meaningful insights on the genetic interactions and cellular makeup of our data.

We call our method the ‘Cartesian linear Gaussian additive noise model’, or $\boxplus$-LGAM for short. Our method is a type of additive noise model [5].

## Background: Kronecker-structured covariance estimation

Broadly, Kronecker-structured covariance estimation aims to estimate covariance (and related quantities) when the covariance matrix can be factorized according to some function. The most basic such model is the matrix normal distribution $\mathcal{M}\mathcal{N}$. Letting $\textrm{vec}\left[ \textbf{X}\right]$ be the vector obtained by stacking the columns of a matrix, and $\otimes$ be the Kronecker product, the matrix normal distribution is:

$$\begin{aligned}& \textbf{X} \sim \mathcal{M}\mathcal{N}\left( \textbf{M}, \boldsymbol{\Sigma }_\textrm{cells}, \boldsymbol{\Sigma }_\textrm{genes}\right) \qquad \\ \iff \textrm{vec}\left[ \textbf{X}\right] & \sim \mathcal {N}\left( \textrm{vec}\left[ \textbf{M}\right] , \boldsymbol{\Sigma }_\textrm{cells} \otimes \boldsymbol{\Sigma }_\textrm{genes}\right) \end{aligned}$$

The factor matrices $\boldsymbol{\Sigma }_\textrm{cells}, \boldsymbol{\Sigma }_\textrm{genes}$ represent the covariances along their respective axes, and their Kronecker product represents the covariance structure of matrix elements. Covariance matrices imply a network structure over the genes (and cells): if $\boldsymbol{\Sigma }^{(genes)}_{ij} \ne 0$, then genes *i* and *j* are correlated, and hence connected in the correlation network.

### Example 1

The correlation of PAX5 and CD19 is found in $\boldsymbol{\Sigma }_{\textrm{genes}}$, the correlation of cell A and cell B is found in $\boldsymbol{\Sigma }_{\textrm{cells}}$, and the correlation between the expression of PAX5 in cell A and the expression of CD19 in cell B is found in $\boldsymbol{\Sigma }_{\textrm{cells}}\otimes \boldsymbol{\Sigma }_{\textrm{genes}}$.

Rather than encoding Kronecker structure on the covariance matrix, it is often advantageous to encode it on the inverse covariance matrix (the ‘precision’ matrix). For Gaussian data, the sparsity structure of the precision matrix encodes the graph of ‘conditional dependencies’, which tend to be more meaningful than correlation.

### Example 2

In a dataset capturing ice cream sales, shark attacks, and seasonality, ice cream sales and shark attacks will be correlated (as both increase in the summer, due to heat and beach visits). However, they will be conditionally independent: once one conditions over seasonality, the correlation disappears.

Various structures have been considered on precision matrices (Kronecker product, Kronecker sum [6], squared Kronecker sum [7, 8], and the strong product [9]). Such structures have also been imposed on the Laplacian of the graph of conditional dependencies [10]. For a more complete picture, we refer the reader to the excellent review [11].

Our model is similar to the squared Kronecker sum approach, although we optimize over directed acyclic graphs (DAGs) and they optimize over symmetric matrices. The Kronecker sum, denoted $\oplus$, is defined as $\textbf{A} \oplus \textbf{B} = \textbf{A}\otimes \textbf{I} + \textbf{I}\otimes \textbf{B}$. The Kronecker sum is interpretable as the *Cartesian product* ($\boxplus$) of the gene and cell graphs encoded by $\textbf{A}$ and $\textbf{B}$ (denoted $\mathfrak {A}$ and $\mathfrak {B}$); the graph of $\textbf{A} \oplus \textbf{B}$ is $\mathfrak {A} \boxplus \mathfrak {B}$. Imposing a Cartesian product structure is biologically meaningful; it corresponds to the assumption that genes can only directly regulate other genes in the same cell.

### Example 3

Suppose there are two cells and two genes of interest, PAX5 and CD19. In such a dataset, there are four different values being measured: PAX5 and CD19’s expressions in the first cell, and PAX5 and CD19’s expressions in the second.

The Cartesian product assumption states that the expression of PAX5 in the first cell may be responsible for the level expression of CD19 *in that same cell*, but that it cannot be (directly) responsible for the level of expression of CD19 in the other cell. The level of PAX5 in both cells are allowed to be directly correlated (and likewise for CD19 in both cells); thus, indirect interactions may occur:

$$\begin{aligned} \textrm{PAX5}_\text {cell 1}\rightarrow \textrm{PAX5}_\text {cell 2}\rightarrow \textrm{CD19}_\text {cell 2} \end{aligned}$$

It is only direct relationships that are forbidden.

Kronecker-structured problems often suffer from non-identifiability due to an underdetermined parameterization. For Kronecker sums (and hence our model), $\textbf{A} \oplus \textbf{B} = \left( \textbf{A} + c\textbf{I}\right) \oplus \left( \textbf{B} - c\textbf{I}\right)$ for any *c*. Thankfully, this non-identifiability does not affect the sparsity structure of $\textbf{A}, \textbf{B}$ - only their diagonals. As we are only interested in the sparsity structure, this non-identifiability is not an issue.

## Methodology: the Cartesian additive noise model

In this paper, we will assume that our scRNA-seq dataset takes the form of a matrix $\textbf{X}$ with $d_{\textrm{cells}}$ cells and $d_{\textrm{genes}}$ genes (see the Tensor-variate datasets section for a tensor-variate generalization). We then model that our dataset abides by the following generative process with parameters $\{\textbf{C}_i\}$:

1 $$\begin{aligned} \textbf{X} & = \textbf{C}_{\textrm{cells}}\textbf{X} + \textbf{X}\textbf{C}_{\textrm{genes}}^T + \textbf{E} \nonumber \\ \textbf{E}_{ij} & \sim _{i.i.d.} \mathcal {N}\left( 0, 1\right) \end{aligned}$$

This is a generalization of the common *linear Gaussian additive noise model* (LGAM), for which $\textbf{X} = \textbf{C}\textbf{X} + \textbf{E}$. We can also write the LGAM as:

LGAM $$\begin{aligned} x_0 & = \epsilon _0 \nonumber \\ x_1 & = c_{10}x_{0} + \epsilon _1 \nonumber \\ \vdots & \nonumber \\ x_{N} & = c_{N0}x_0 + \cdots + c_{N,N-1}x_1 + \epsilon _N \end{aligned}$$

LGAM is a generative causal model. Let $\mathfrak {C}$ be the causal graph, i.e. $(j \rightarrow i) \in \mathfrak {C}$ means that ‘gene j regulates gene i’. Under the LGAM assumption, knowledge of $\textbf{C}$ yields knowledge of $\mathfrak {C}$: $c_{ij} \ne 0 \iff (j \rightarrow i) \in \mathfrak {C}$ for $i \ne j$. $c_{ii}$ controls the variance.

### Example 4

The causal graph $\{x_0 \rightarrow x_2 \rightarrow x_3$ and $x_1 \rightarrow x_3\}$ is represented as:

$$\begin{aligned} x_0= & \epsilon _0 \\ x_1= & \epsilon _1 \\ x_2= & c_{20}x_0 + \epsilon _2 \\ x_3= & c_{31}x_1 + c_{32}x_2 + \epsilon _3 \end{aligned}$$

Learning causality from observational data is challenging; the gold standard will always be interventional experiments. However, there are so many potential gene-gene interactions that verifying all of them experimentally is prohibitive. Models such as LGAM can be used to shortlist potential candidates for such studies, and allow researchers to operate under plausible conjectures on the gene network in the meantime.

To learn the gene network, LGAM must assume that the cells are independent and identically distributed. Neither assumption is justifiable; cells come in distinct cell types and were sampled from a microenvironment in which they are all interacting. This motivates our model (Eq. 1). Our model allows both the cells and the genes to have their own interactions, captured by $\textbf{C}_{\textrm{cells}}$ and $\textbf{C}_{\textrm{genes}}$. Our model makes four assumptions about the data:

### Assumption 1

The genes (resp. cells) interact *linearly*.

### Assumption 2

The data is Gaussian.

### Assumption 3

Gene/cell dependencies interact via the Cartesian product (see Example 3).

### Assumption 4

$\mathfrak {C}_{\textrm{genes}}$ and $\mathfrak {C}_{\textrm{cells}}$ are directed acyclic graphs (DAGs) with *known order*.

The first two assumptions are not egregious, and often hold approximately even when they fail. In the Simulated scRNA-seq data section, we will see that we still perform well on data that violates these assumptions. The third assumption is the result of the structure of our model; Eq. 1 can be rewritten as:

2 $$\begin{aligned} \textrm{vec}\left[ \textbf{X}\right] & = \left( \textbf{C}_{\textrm{genes}} \oplus \textbf{C}_{\textrm{genes}}\right) \textrm{vec}\left[ \textbf{X}\right] + \textrm{vec}\left[ \textbf{E}\right] \nonumber \\ \textbf{E}_{ij} & \sim _{i.i.d.} \mathcal {N}\left( 0, 1\right) \end{aligned}$$

It is due to this relation that we call our model the *Cartesian Gaussian Additive Noise Model*, or $\boxplus$-LGAM for short. Making an assumption like this is unavoidable; the full space of possible interactions in a scRNA-seq dataset is quite vast. In theory, any gene in any cell could interact with any other gene in any other cell! The problem is equivalent to estimating a covariance matrix from a single sample; without an assumption, this is statistically intractable. The Cartesian assumption is a natural one, and ensures tractability.

Our final assumption is that we know the causal order of genes, i.e. we know whether a gene is upstream or downstream of other genes in regulatory pathways. Unlike our other assumptions, this one is genuinely limiting. Without this assumption, fitting our model corresponds to a non-convex optimization problem. Furthermore, it is known that causal graphs for linear Gaussian data are fundamentally unlearnable beyond what is called the ‘Markov equivalence class’; essentially, there would be multiple equally valid solutions. In the Inferring the ordering section, we present a method to infer causal ordering from the data, and in the Effect of ordering and cyclic edges section we show that using the wrong order does not dramatically deteriorate performance.

### Fitting the model

Given the causal ordering, we can without loss of generality assume $\textbf{C}_\ell$ is lower triangular. For notational simplicity, let $d_{\backslash \ell } = \frac{\prod _{\ell '} d_{\ell '}}{d_\ell }$ and *K* be the number of axes in the data. For matrix-variate datasets, $K=2$; see the Tensor-variate datasets section for examples with larger *K*. Then, define $\textbf{L}_\ell = \sqrt{d_{\backslash \ell }}\left( \frac{1}{K}\textbf{I} - \textbf{C}_\ell \right)$. $\textbf{L}_\ell$ is a natural parameter to consider; it has a 1-to-1 relationship with $\textbf{C}_\ell$ (in particular, it has the same sparsity structure) and corresponds to the ‘Cholesky factor’ of the precision matrix of the data. It is always invertible whenever $\textbf{C}_\ell$ represents a DAG [12] (which is guaranteed by being lower triangular). Finally, by defining $\textbf{L} = \oplus _\ell \frac{1}{\sqrt{d_{\backslash \ell }}}\textbf{L}_\ell$, we have that $\textrm{vec}\left[ \textbf{X}\right] \sim \mathcal {N}\left( \textbf{0}, \textbf{L}^{-T}\textbf{L}\right)$.

Letting $\mathbb {L}^\oplus _{++}$ be the space of all positive-diagonal lower-triangular matrices $\textbf{L}$ that can be decomposed by the Kronecker sum, we can fit our model by finding the maximum likelihood of a normal distribution whose parameters lie in $\mathbb {L}^\oplus _{++}$:

3 $$\begin{aligned} \hat{\textbf{L}} = \textrm{argmin}_{\textbf{L}\in \mathbb {L}_{++}^\oplus } \Vert \textbf{L}\textrm{vec}\left[ \textbf{X}\right] \Vert _2^2 - 2\sum _i \textbf{L}_{ii} \end{aligned}$$

Most real world networks are sparse; we also place an L1 penalty on the off-diagonals ($\lambda _\ell \Vert \textbf{L}^{(\ell )}\Vert _{od}$) to ensure sparse solutions. $\lambda _\ell \Vert \cdot \Vert _{od}$ is frequently used in graphical estimation problems, such as the Graphical Lasso [13]. Additionally, we will see in the Theory: Establishing the efficacy of the method section that, if our data is ‘too lopsided’ ($d_\ell \gg d_{\backslash \ell }$), a solution to this optimization problem is not guaranteed to exist. To counteract this, we add an optional Frobenius-norm penalty as well. Our final optimization problem, below, is thus analogous to ElasticNet [14]:

4 $$\begin{aligned} \hat{\textbf{L}} = \textrm{argmin}_{\textbf{L}\in \mathbb {L}_{++}^\oplus } \Vert \textbf{L}\textrm{vec}\left[ \textbf{X}\right] \Vert _2^2 - 2\sum _i \textbf{L}_{ii} + \sum _\ell \left( \rho _\ell \Vert \textbf{L}^{(\ell )}\Vert _F^2 + \lambda _\ell \Vert \textbf{L}^{(\ell )}\Vert _{od}\right) \end{aligned}$$

### Tensor-variate datasets

Most omics datasets are matrix-variate: a scRNA-seq dataset has genes and cells, scATAC-seq has peaks and cells, bulk RNA has samples and genes, etc. In these cases, the matrix-variate $\boxplus$-LGAM will suffice. However, some datasets are more complicated. A longitudinal study of gene expression in a cohort of patients would naturally lend itself to a tensor-variate approach: the dataset $\mathcal {X}$ would have three axes, one for time, one for genes, and one for patients.

Our model is readily generalized to such a scenario. Tensor notation can be complex; we refer the reader to [15] for a full overview. Here, we need the concept of the $\ell$-mode product $\times _\ell$, which intuitively corresponds to multiplying a tensor along the axis $\ell$. The formal definition can be hard to parse; $[\mathcal {X} \times _\ell \textbf{M}]_{i_1i_2\cdots i_\ell \cdots i_K} = \sum _{j_\ell } \mathcal {X}_{i_1i_2\cdots i_{\ell -1}j_\ell i_{\ell +1} \cdots i_K} \textbf{M}_{j_\ell i_\ell }$. For matrices, $\textbf{A}\times _1\textbf{B} = \textbf{B}^T\textbf{A}$ and $\textbf{A} \times _2 \textbf{B} = \textbf{A}\textbf{B}$.

5 $$\begin{aligned} \mathcal{X} &= \left(\sum_\ell^K\mathcal{X} \times_\ell \mathbf{C}_\ell^T\right) + \mathcal{E} \\ \mathcal {E}_{\textbf{i}} &\sim _{i.i.d.} \mathcal {N}\left( \textbf{0}, \textbf{I}\right) \end{aligned}$$

Other than having more parameters to learn, the model is functionally identical to the matrix-variate case; it makes the same assumptions and is fit in the same manner.

## Theory: Establishing the efficacy of the method

In the Methodology: the Cartesian additive noise model section, we made four assumptions:

### Assumption 1

The genes (resp. cells) interact *linearly*.

### Assumption 2

The data is Gaussian.

### Assumption 3

Gene/cell dependencies interact via the Cartesian product (see Example 3)

### Assumption 4

$\mathfrak {C}_{\textrm{genes}}$ and $\mathfrak {C}_{\textrm{cells}}$ are directed acyclic graphs (DAGs) with *known order*.

These govern whether or not our model is correct to use on a dataset, but do not allow us to quantify *how well* our model can be expected to do. For this, we need to make two more assumptions.

### Assumption 5

Our dataset is not too lopsided, i.e. the largest axis $d_\textrm{max}$ is not much greater than the other axes. Formally, $K - 1 + \prod _\ell d_\ell \ge \sum _\ell \frac{d_\ell ^2-d_\ell }{2}$, but the following are simpler sufficient conditions:

- For matrix-variate data, $d_{\textrm{max}} \le d_{\textrm{min}} + \frac{1+\sqrt{9+8d_{\textrm{min}}}}{2}$ (both necessary and sufficient)
- For tensor-variate data, $d_{\backslash \textrm{max}}\ge \frac{K}{2}d_\textrm{max}$

### Assumption 6

Our dataset is $\alpha$-sub-exponential, with $\alpha \in (0, 2]$.

### Definition

($\alpha$-sub-exponentiality) A distribution $\mathcal {D}$ is $\alpha$-sub-exponential if for $\alpha > 0$, some constant *c* and all $t>0$, $\mathbb {P}_{x\sim \mathcal {D}}\left( \left| x - \mathbb {E}\left[ x\right] \right| \ge t\right) \le 2e^{\frac{-t^\alpha }{c}}$. In other words, $\alpha$-sub-exponential distributions have light tails.

### Example 5

All sub-Gaussian distributions, including the Gaussian distribution and all bounded distributions, are $(\alpha \ge 2)$-sub-exponential. The Poisson and negative binomial distributions are $(\alpha =1)$-sub-exponential.

### Example 6

The Cauchy, log-normal, and t distributions are **not **$\alpha$-sub-exponential.

Note that the $\alpha$-sub-exponentiality assumption is more general than the Gaussian assumption, and encompasses many distributions typically used to model scRNA-seq data (such as the Poisson and negative binomial distributions). Making this assumption allows us to prove error bounds on our model, described by Theorem 1.

The statement of Theorem 1 is dense. The details may not be of interested to every user, so we will first summarize the essence of the statement. The theorem is proven in the supplementary material using standard high-dimensional statistics techniques, including the generalized Hanson-Wright inequalities of [16].

### Theorem

(Theorem 1, paraphrased) With exponentially high probability, we can bound the error of our model in terms of our regularization parameters $\rho$, $\lambda$. This gives us faith in our model’s utility.

### Theorem 1

Let $\lambda _\ell = \lambda$ and $\rho _\ell = \rho$ for each axis $\ell$. Assume our data is $\alpha$-sub-exponential with $\alpha \in (0, 2]$. Suppose the largest Frobenius norm of any factor of the oracle solution^1^$\textbf{L}^*_\rho$ is $R_F$, the total amount of nonzero elements of the factors of the regularized oracle solution is *s*, and $\mu$ is the strong convexity constant of Eq. 3. Let *p* be a parameter we choose to control the probability of success, and $\beta$ be defined shortly. Let $\lambda \asymp \max _\ell {\sqrt{\frac{\left( p\log d_\ell \right) ^\beta }{d_{\backslash \ell }}} + \sum _{\ell '\ne \ell } \sqrt{\frac{\left( p\log d_\ell \right) ^\beta }{d_\ell d_{\ell '}}}}$, then with probability at least $1-\sum _\ell 2^{1-p\log d_\ell }$:

$$\begin{aligned} \Vert \hat{\textbf{L}} - \textbf{L}^*\Vert _{F,\oplus } \le \frac{2K\rho }{\mu }R_F + \frac{3\lambda \sqrt{s}}{\mu +\rho } \end{aligned}$$

For matrix-variate data, let $\lambda \asymp \max \left( \sqrt{\frac{\left( \log d_1\right) ^\beta }{d_2}}, \sqrt{\frac{\left( \log d_2\right) ^\beta }{d_1}}\right)$.

$\beta$ is either 1 or $\frac{4}{\alpha }$. $\beta =1$ corresponds to the ‘typical regime’, and $\beta =\frac{4}{\alpha }$ applies when *p* is chosen to be very large. We derive an expression for the boundary between the two regimes in the supplementary material, but, for reasonable^2^*p*, $\beta =1$ should suffice.

### Corollary 1

Suppose that the smallest value of the oracle solution $\textbf{L}^*$ is larger than $\frac{2K\rho }{\mu }R_F + \frac{3\lambda \sqrt{s}}{\mu +\rho }$. Then (under Theorem 1’s assumptions), our model is ‘*sparsistent*’: with exponentially high probability, the support, and hence the true network $\mathfrak {C}$, is recovered correctly.

Our other assumption concerns the ‘lopsidedness’ of our data. It is natural to expect some kind of restriction here: suppose we had 1,000,000 cells and 5 genes. How are we possibly going to estimate a cell network which has $\approx \frac{10^{12}}{2}$ parameters from only 5 genes? One axis cannot be too much larger than the other, or else it will be too difficult to estimate that axis’ network. This is not as limiting as it first seems: we will see that, if we choose $\rho _\ell > 0$ in Eq. 4, then lopsidedness is not a concern.

### Theorem 2

(Lopsidedness Theorem) As long as the dataset is not too lopsided and the true distribution has positive definite covariance, then Eq. 4 defines a function that is almost surely strongly convex when every $\rho _\ell = 0$. Equation 4 is always strongly convex when $\rho _\ell > 0$.

### Corollary 2

When the conditions of Theorem 2 are satisfied, there exists a unique solution to Eq. 4.

The proof relies on Cover’s Theorem [17], and is given in the supplementary material. Even when Theorem 2 fails, a solution can still exist with high probability.

### Theorem 3

(Excess Lopsidedness Theorem) Let $d_\forall = \prod _\ell d_\ell$ and $D = \sum _\ell \frac{d_\ell ^2-d_\ell }{2} - K + 1 - d_\forall$, and suppose $D > 0$; in other words, *D* represents the ‘excess lopsidedness’ of the data. For $(d_1, d_1+\delta )$-shaped (matrix-variate) data, $D = \frac{\delta ^2-\delta }{2} - (d_1+1)$.

With probability $1 - \frac{1}{2^{d_\forall -1}}\sum _{i=0}^{\textrm{min}\left( D, d_\forall \right) )-1}{d_\forall -1 \atopwithdelims ()i}$ a solution exists when $\rho _\ell =0$ to Eq. 4.

### Example 7

Assume Frobenius penalties $\rho _\ell$ of zero. For an arbitrary $100 \times 114$ dataset, Eq. 4 almost surely has a solution. For a $100 \times 263$ dataset it has at least a 50% chance of having a solution. In all scenarios in which $\rho _\ell > 0$, a solution exists.

### Example 8

Tensor-variate data is permissive. A solution almost surely exists for $100 \times 100 \times 6000$ and $100 \times 100 \times 100 \times 500000$ datasets.

In the Simulated scRNA-seq data section, we will see that our model is able to perform well on lopsided data when $\rho _\ell > 0$. The dataset in question has 640 cells and 11 genes, in which case Eq. 4 has *no solution* when all $\rho _\ell$ are 0 (Theorem 3).

### Inferring the ordering

The most egregious assumption we make is knowledge of the ordering. When the order is unknown, we could change Eq. 4 to optimize over the space of all DAGs, rather than lower-triangular matrices. However, this problem is NP-hard [18]. In this section, we’ll show that, under certain conditions, the ordering can be recovered from the data.

For the LGAM model, the ordering of the variables can be inferred from the variance of the data.

#### Theorem

(Various Authors) Let $\textbf{x}$ come from the LGAM model. Let $i < i'$ denote that there is a path from *i* to $i'$ in the DAG, i.e. *i* comes earlier in the ordering. Then, under various conditions [19, 20], we have that:

$$\begin{aligned} i< i' \implies \textrm{Var}\left[ \textbf{x}_i\right] < \textrm{Var}\left[ \textbf{x}_{i'}\right] \end{aligned}$$

One such condition is that the diagonals of each $\textbf{C}_\ell$ are homogeneous.

In fact, any such result like this can be adapted to fit our $\boxplus$-LGAM model.

#### Theorem 4

Let $\mathcal {X}$ come from the $\boxplus$-LGAM model. Under various conditions [19, 20], we have that:

$$\begin{aligned} i< i' \implies \mathbb {E}\left[ \sum \limits _\textbf{j} \mathcal {X}^2_{i\textbf{j}}\right] < \mathbb {E}\left[ \sum \limits _\textbf{j} \mathcal {X}^2_{i'\textbf{j}}\right] \end{aligned}$$

One such condition is that the diagonals of each $\textbf{C}_\ell$ are homogeneous.

#### Example 9

Let’s assume that $\textrm{diag}\left[ \textbf{C}_{\ell }\right] = a_\ell \textbf{I}$ for some constants $\{a_\ell \}$. This is equivalent to assuming that every gene has the same variance and every cell has the same variance after conditioning out the effect of other genes/cells. Then one should order the terms (in ascending order) by their empirical variance.

In the real world, gene variances are unlikely to be homogeneous. Other, more complicated assumptions on the variance will suffice for Theorem 4 [20]. Additionally, the variance heuristic can be combined with domain knowledge; one can first order genes by whether or not they are transcription factors before applying this heuristic, for example. In the Effect of ordering and cyclic edges section, we show that, even if the chosen ordering is *dramatically* wrong, the recovered network still maintains a good degree of accuracy.

## Results

It is often hard to validate graphical models, given that we rarely have access to a ground truth network. We’ll analyze our performance on two types of synthetic data: that which we generate ourselves from simple distributions to ensure a given structure (Synthetic data section) and a simulated scRNA-seq dataset from the literature (Simulated scRNA-seq data section). We’ll call the former ‘synthetic’ and the latter ‘simulated’, to reflect the fact that the latter dataset is an expert-curated simulation of real-world data. We also investigate the effects of getting the ordering wrong (in the Effect of ordering and cyclic edges section).

### Synthetic data

Each of the synthetic datasets we will experiment with are generated from our $\boxplus$-LGAM model. We focus on in-distribution data in this section to get an understanding of the idealized performance of our model; out-of-distribution data is the focus of the Simulated scRNA-seq data section. We do not explore the effect of $\rho$ here, but will in the Simulated scRNA-seq data section. We let our L1 penalty ($\lambda$) be the same for all axes.

**Table 1:** Results on synthetic data, ordered by problem difficulty

Dimension	AUC-PR			MCC		
	LGAM	$\boldsymbol{\boxplus }$-LGAM	GmGM	LGAM	$\boldsymbol{\boxplus }$-LGAM	GmGM
$100_{\textrm{ER}}\times 50_{\mathrm {AR(1)}}$	0.778	**0**.**823**	0.247	0.726	**0**.**781**	0.369
$40_{\textrm{ER}}\times 40_{\textrm{ER}} \times 40_{\textrm{ER}}$	0.791	**0**.**801**	0.648	0.726	**0**.**756**	0.674
$60_{\textrm{ER}}\times 60_{\textrm{ER}} \times 60_{\textrm{ER}}$	0.758	**0**.**775**	0.688	0.684	**0**.**707**	0.650
$50_{\textrm{ER}}\times 50_{\mathrm {AR(1)}}$	0.680	**0**.**762**	0.370	0.670	**0**.**715**	0.552
$50_{\textrm{ER}}\times 100_{\mathrm {AR(1)}}$	0.468	**0**.**598**	0.138	0.600	**0**.**676**	0.309
2.5% $100_{\textrm{ER}}\times 100_{\textrm{ER}}$	0.417	**0**.**427**	0.272	0.508	0.504	**0**.**527**
$100_{\mathrm {AR(1)}}\times 50_{\textrm{ER}}$	0.367	**0**.**386**	0.288	0.344	**0**.**346**	0.275
$50_\mathcal {I}\times 50_{\textrm{ER}}$	0.348	**0**.**354**	0.316	**0**.**335**	0.330	0.328
$50_{\textrm{ER}}\times 50_{\textrm{ER}}$	0.315	0.328	**0**.**345**	0.291	0.321	**0**.**333**
5% $100_{\textrm{ER}}\times 100_{\textrm{ER}}$	0.270	**0**.**316**	0.272	0.293	0.347	**0**.**383**
$100_{\textrm{ER}}\times 50_{\textrm{ER}}$	0.297	**0**.**308**	0.276	0.313	**0**.**333**	0.283
10% $100_{\textrm{ER}}\times 100_{\textrm{ER}}$	0.250	**0**.**279**	0.239	0.241	**0**.**264**	0.237
$50_{\textrm{ER}}\times 100_{\textrm{ER}}$	0.209	**0**.**226**	0.175	0.188	**0**.**207**	0.152
$50_{\mathrm {AR(1)}}\times 100_{\textrm{ER}}$	**0**.**197**	0.190	0.164	**0**.**175**	0.159	0.133

We report both the area under the precision-recall curve (AUC-PR) and the best Matthew’s correlation coefficient (MCC) achieved by the methods. Results are reported on the second axis of the dataset - for the lopsided $100 \times 50$ and $50 \times 100$ datasets, note that the first-axis performance of $50\times 100$ is the same as the second-axis performance of $100 \times 50$. All ground truth graphs were 10% dense, except if labeled 5% dense (matching Scenario 2), or 2.5% dense (matching Scenario 3). The subscript of an axis indicates the type of graph used (ER is Erdös-Renyi, AR(1) is autoregressive, and $\mathcal {I}$ is independent/an empty graph). The best result for each task is given in bold

We investigate many different scenarios, based on the size, sparsity and lopsidedness of the dataset. Typically, our ground truth is generated to be an Erdös-Renyi graph (i.e, each potential edge has a constant probability to be present in the graph, independent of other edges), although we also look at AR(1) graphs (in which the graph is a chain of vertices; vertex *i* is only connected to vertices $i\pm 1$). These graphs were chosen as they represent two extremes; Erdös-Renyi graphs are naturally unstructured whereas AR(1) graphs have a simple and consistent structure. We summarize results in Table 1, using standard LGAM and GmGM [21] as baselines. GmGM estimates the (symmetric) graph of conditional dependencies (as in the Background: Kronecker-structured covariance estimation section) rather than a directed graph, but it is also based on the Cartesian product.

Our method performs very well for tensor-variate and/or AR(1) data. In most cases, our method outperformed the baselines. Our method achieved worse results on highly-sparse-graph data compared to the baseline. This is not surprising; when the sample graph is highly sparse, the dataset is very close to being independent. When the samples truly are independent, LGAM suffices and our model may overfit. GmGM sometimes does best, but is typically much worse than LGAM and $\boxplus$-LGAM.

When comparing the effect of dataset size on performance, we considered three different scenarios, going from a size of $50\times 50$ to $100\times 100$. These correspond to three different scenarios for the amount of edges *s* in the true growth.

#### Scenario 1

The percentage of extant edges stays the same; $s=O(d_\ell ^2)$.

#### Scenario 2

The average degree of nodes stays the same; $s=O(d_\ell )$.

#### Scenario 3

The amount of total edges stays the same; $s=O(1)$.

According to Theorem 1, the error for a $d\times d$ dataset should grow as $O(\sqrt{s})$; thus, we’d expect the performance to decrease in Scenario 1 and increase in Scenario 3; this is exactly what we see.

#### Runtime and asymptotic complexity

We fit our model with accelerated proximal gradient descent; thus, there are two components to the runtime (the amount of iterations, and the cost of gradient computation). Assuming the lopsidedness condition is satisfied (Theorem 2), the problem is strongly convex and so will exhibit linear convergence. We derive the computational complexity of gradient computation in Section B of the supplementary material. The full expression is complicated, so we also give a simplified computational complexity in terms of the number of axes *K* and largest axis $d_\textrm{max}$:

Full complexity $$\begin{aligned} O\left( \sum _\ell \left( d_\ell ^3 + K\sum _{\ell '\ne \ell }d_{\ell '}^2\prod _{\ell ''\ne \ell '}d_{\ell ''}\right) + \prod _\ell d_\ell \right) \end{aligned}$$

Simplified $$\begin{aligned} O\left( K^2d_{\textrm{max}}^{K+1}\right) \end{aligned}$$

Matrix-variate data is the most common use-case; in this scenario, the complexity is cubic. Due to linear convergence, the time to reach an error of $\epsilon$ is $O(K^2d_\textrm{max}^{K+1}\log \frac{1}{\epsilon })$. This is standard for Kronecker-sum-structured models, and is as good as could be expected without making further assumptions; the complexity comes from the cubic cost of computing matrix multiplication. The space complexity comes from the cost of holding these matrices in memory; the largest intermediate matrix is of size $O(d_\textrm{max}^2)$.

It should be noted that a naïve implementation of this method would have much worse complexity; in particular, the space complexity alone would be $O(d^{2K})$ due to the cost of storing $\textbf{L}\in \mathbb {L}^\oplus _{++}$. We are able to avoid this by exploiting properties of the Kronecker sum to express the problem in terms of matrix multiplications involving only the factor matrices $\textbf{L}^{(\ell )}$.

Empirically, we measured our runtime over various scenarios on synthetic data; the results are given in Table 2. In general, our method takes around four times longer to run than LGAM. Given that $\boxplus$-LGAM produces estimates *for each axis*, whereas LGAM needs to be run independently for each axis, it is also relevant to note that on a per-axis level $\boxplus$-LGAM is about half as fast as LGAM for matrix variate data (and slightly faster for tensor data). Empirical runtimes increase at a faster rate than asymptotics would suggest.

**Table 2:** Runtimes (seconds) on synthetic Erdös-Renyi data of various shapes

Algorithm	Matrix-variate					3-Axis		4-Axis
	$\boldsymbol{25}_{\textbf{ER}}^{\boldsymbol{2}}$	$\boldsymbol{50}_{\textbf{ER}}^{\boldsymbol{2}}$	$\boldsymbol{75}_{\textbf{ER}}^{\boldsymbol{2}}$	$\boldsymbol{100}_{\textbf{ER}}^{\boldsymbol{2}}$	$\boldsymbol{100}_{\mathbf {AR(1)}}^{\boldsymbol{2}}$	$\boldsymbol{25}_{\textbf{ER}}^{\boldsymbol{3}}$	$\boldsymbol{50}_{\textbf{ER}}^{\boldsymbol{3}}$	$\boldsymbol{25}_{\textbf{ER}}^{\boldsymbol{4}}$
$\boxplus$-LGAM	0.05	0.83	7.1	34	36	2.8	172	134
LGAM	0.02	0.23	1.8	8.8	7.9	1.1	55	40
Increase over LGAM	$\times 2.0$	$\times 3.8$	$\times 4.0$	$\times 3.9$	$\times 4.6$	$\times 2.5$	$\times 3.1$	$\times 3.3$
Per-axis increase	$\times 0.99$	$\times 1.9$	$\times 2.0$	$\times 1.9$	$\times 2.3$	$\times 0.82$	$\times 1.0$	$\times 0.83$

Runtimes vary by the strength of the regularization parameter; here we present the average runtime over 10 runs with logarithmically spaced L1 penalties in the range $[10^{-2}, 10^2]$, until convergence to a tolerance of $10^{-3}$

There is scope to improve the speed of the algorithm, for example by the use of second-order methods. GmGM achieved stronger scalability than other analogous methods by using thresholding rather than an L1 penalty; such an approach could be used here as well. For this comparison, we fit LGAM in the same way we fit $\boxplus$-LGAM (proximal accelerated gradient descent). More advanced methods of fitting LGAM could be translated to more advanced methods of fitting $\boxplus$-LGAM as well.

### Effect of ordering and cyclic edges

When the ordering is unknown, we’d like to know how much the choice of ordering is likely to affect the sparsity structure of the recovered graph. We empirically test the effect of getting the ordering wrong on synthetic data, in Table 3. These results demonstrate that getting the ordering wrong does not dramatically affect structure recovery. If the ordering is unknown, heuristics and theorems that allow us to approximate the ordering, such as Theorem 4, are helpful, but not necessary - even with a completely random ordering, structure recovery capability is mostly preserved. However, if the true underlying DAG is cyclic, then the performance of both both LGAM and $\boxplus$-LGAM can be significantly impacted; this is seen in Table 3. The degree of impact is governed by the strength of cyclicity; there was minimal impact when the backwards-pointing edges were fewer in number than forwards-pointing.

**Table 3:** Results on synthetic data; the same dataset is used for each, with only the ordering changed

Ordering	AUC-PR		MCC	
	LGAM	$\boldsymbol{\boxplus }$-LGAM	LGAM	$\boldsymbol{\boxplus }$-LGAM
True Ordering	0.778	**0**.**823** ($\uparrow$6%)	0.726	**0**.**781** ($\uparrow$8%)
20% Shuffled	0.776	**0**.**824** ($\uparrow$6%)	0.726	**0**.**777** ($\uparrow$7%)
50% Shuffled	0.730	**0**.**773** ($\uparrow$6%)	0.709	**0**.**751** ($\uparrow$6%)
100% Shuffled	0.728	**0**.**798** ($\uparrow$10%)	0.745	**0**.**768** ($\uparrow$3%)
Strongly Cyclic	0.364	**0**.**418** ($\uparrow$14%)	0.376	**0**.**424** ($\uparrow$12%)
Weakly Cyclic	0.734	**0**.**777** ($\uparrow$5%)	0.663	**0**.**708** ($\uparrow$6%)

We report both the area under the precision-recall curve (AUC-PR) and the best Matthew’s correlation coefficient (MCC). Results are reported on the second axis of the dataset. All tests were run on the $100_\textrm{ER}\times 50_{\mathrm {AR(1)}}$ Gaussian test from Table 1. An ordering is x% shuffled if we randomly shuffled the first x% of the ordering. For the strongly cyclic test, we replaced each true graph $\textbf{L}$ with $\textbf{L}+\textbf{L}^T$ before generating the data. For the weakly cyclic graph, we generated a second 2.5% dense graph $\textbf{L}_2$, using $\textbf{L}+\textbf{L}_2^T$ as the ground truth. The best result for each task is given in bold

### Simulated scRNA-seq data

**Fig. 1:**
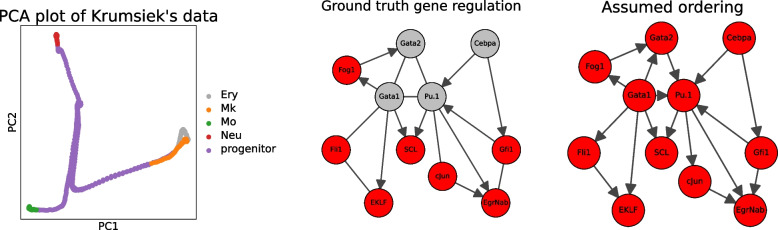
(Left) The first two PCA components of the Krumsiek dataset, demonstrating clear developmental trajectories. (Center) The regulatory network used to generate the dataset. Some edges in their network were bi-directional; these have been represented as undirected edges. Gata1, Gata2, Pu.1, and Cebpa have ‘self loops’. (Right) The ground truth graph with our assumed ordering

In this section, we apply our method to the simulated dataset of [22]. The dataset in question (the ‘Krumsiek dataset’) was generated from a literature-curated regulatory network of 11 genes involved in blood cell differentiation. The regulatory network is not acyclic: it contains some loops (Fig. 1(Center)).

The dataset contains 640 cells, which are labeled by cell type (erythrocyte, megakaryocyte, monocyte, neutrophil, and progenitor). The dataset has a notable ‘developmental structure’: progenitor cells differentiate into the other cells; in Fig. 1(Left) we can see this developmental process manifest in PCA-space.

We compare our performance against the LGAM baseline. The task for the gene axis is clear; we wish to know how well our method recovers the structure of the ground truth graph. For the cell axis, this is less clear. Given that we have access to cell labels, we evaluate how well cells of the same type cluster in our graphs. We are also interested on how well, qualitatively, our graphs recover the developmental structure of the dataset.

#### Establishing causal ordering

To determine if a gene comes before another, we apply the following three rules in order of priority:

1. Must gene A come before gene B due to prior knowledge?
2. If a gene self-regulates, it comes before genes that do not.
3. Use Theorem 4 to determine the ordering.

As the ground truth gene graph was constructed by [22] using the academic literature, we’ll assume knowledge of the orderings that are entailed by the graph. The ground truth graph contains both uni-directional and bi-directional edges, so not every ordering is known. For example, the graph contains Gata1 $\rightarrow$ Fog1 and Gata1 $\leftrightarrow$ Fli1. Thus, Gata1 must come before Fog1, but we do not know the ordering of Gata1 and Fli1. We will also assume knowledge about which genes self-regulate (Gata1, Gata2, Pu.1, Cebpa). These genes are likely to have higher variance due to this, which would interfere with Theorem 4. After following this process, we get the ordering:

$$\begin{aligned} \textrm{Gata1}&\rightarrow \textrm{Fog1}\rightarrow \textrm{Gata2}\rightarrow \textrm{Cebpa}\\&\rightarrow \textrm{Gfi1}\rightarrow \mathrm {Pu.1}\rightarrow \textrm{Fli1}\\&\rightarrow \textrm{EKLF}\rightarrow \textrm{cJun}\rightarrow \textrm{EgrNab}\rightarrow \textrm{SCL} \end{aligned}$$

What about the ordering of the cells? We order our cells by their ‘pseudotime’. Pseudotime is a measure of how far along its developmental trajectory a given cell is. scRNA-seq datasets contain only a single snapshot in time (hence the ‘pseudo’ in ‘pseudotime’), but not every cell ends up developing at the same rate. Thus, scRNA-seq datasets can often capture the developmental trajectory of a cell type (in this case, the differentiation of progenitors into the other cell types).

To estimate pseudotime, we used PAGA [23] to construct the coarse, large-scale structure of development and DPT [24] to assign each cell a distance along this trajectory. We chose this method as it is the standard workflow in the scanpy package [25], which we used for our experiments.

#### Choosing the parameters

For our $\boxplus$-LGAM model, we first grid-searched over the two L1 penalties $\lambda _1, \lambda _2$, with fixed $\rho$. As we don’t have a quantitative metric for the cell graph’s performance, we used the performance (Matthew’s correlation coefficient) on the gene graph to rank results. We then picked the best-performing result, and performed an extended grid-search over $\rho$. For the baseline, we grid-searched over $\lambda$ and $\rho$. See Figure A in the supplementary material for grid search results, and Fig. 3(Left) for the best baseline gene graph.

**Fig. 2:**
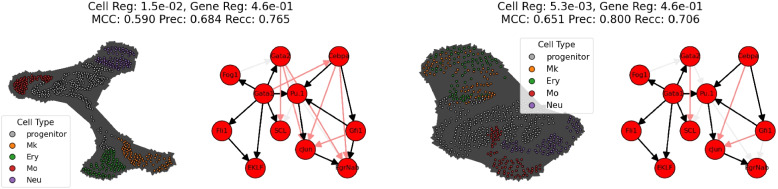
(Left) A gene graph arising from our $\boxplus$-LGAM’s grid search. Chosen for display as its cell graph visually appears the most similar to the known developmental pathways of these cell types. (Right) The best gene graph arising from our $\boxplus$-LGAM model’s grid search. (All) Black edges are true positives, near-white edges are false negatives, pale red edges are false positives

**Fig. 3:**
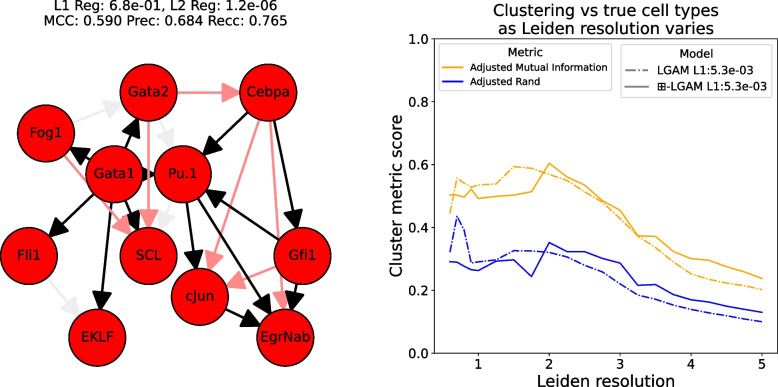
(Left) The best gene graph arising from the baseline LGAM’s grid search. Black edges are true positives, near-white edges are false negatives, pale red edges are false positives. (Right) Clustering metrics comparing the best $\boxplus$-LGAM results to the best baseline LGAM result

To investigate the ability of the cell graph to qualitatively capture the developmental structure, we looked at the three best performing cell graphs from the original grid search, as well as the best performing cell graph from the extended grid search. Figure 2(Left) displays the one we, based subjectively on its visual appearance, felt captured the known cellular development pathway the best. For a more objective measure, we evaluated cluster quality when using the Leiden clustering algorithm [26], given in Fig. 3(Right).

One benefit of our model is that the gene and cell objectives are linked; if we have a metric for gene graph performance, we can optimize for that and produce a corresponding cell graph. For standard LGAM, we have no way of doing so. To be able to compare to a baseline, we ran LGAM three times, once for each of the top 3 cell parameters from our $\boxplus$-LGAM grid search.

The results are favorable to our model; our best gene graph achieved an MCC of 0.651, compared to 0.590 for the baseline. By optimizing only over gene graph performance, we automatically learned cell graphs as well, which in most cases outperformed the baseline in clustering quality measured by adjusted mutual information and adjusted Rand scores. While learning the graphs based on *both* cell and gene metrics would likely lead to better cell graphs than those based just on gene metrics, the fact that we only need one metric to learn both graphs is certainly a benefit; single-axis models like LGAM need a metric for each graph they learn.

#### Does domain knowledge help?

In the Establishing causal ordering section, we established causal ordering using three steps, two of which were meant to capture a practitioner applying domain knowledge in the process. Here, we will explore what happens if one does not have any domain knowledge. In particular, here we only use Theorem 4 to estimate the causal ordering. This leads to the following ordering:

$$\begin{aligned} \textrm{Fli1}&\rightarrow \textrm{Fog1}\rightarrow \textrm{EKLF}\rightarrow \textrm{Gata2}\\&\rightarrow \textrm{Gfi1}\rightarrow \textrm{cJun}\rightarrow \textrm{EgrNab}\\&\rightarrow \textrm{Cebpa}\rightarrow \mathrm {Pu.1}\rightarrow \textrm{Gata1}\rightarrow \textrm{SCL} \end{aligned}$$

We then performed a grid search over the parameters as before (Figure B in the supplementary material). The best gene MCC for $\boxplus$-LGAM was 0.55, compared to LGAM’s 0.51 over the same grid and ordering. These are both lower than the previous ordering, showing that we did get a benefit by taking into account domain knowledge.

### A demonstration on simple tensor-variate data

This paper focuses on matrix-variate data as that is by far the most common form of structured omics data. However, we have been careful to keep our methodology and theoretical results general in case the need for tensor-variate methods arise. In this section, we briefly consider a simplified real-world example to demonstrate the use of our method for such data. For this we use the duck image from the COIL-20 dataset [27]. The ‘duck image’ is a 72-frame series of $128 \times 128$ pixel images of a rotating rubber duck. The frames have a clear causal structure: each frame should be caused by the previous frame. As there is a 360 degree rotation, the natural graph is cyclic. To account for this, we dropped the last half of the frames, and then further downsampled to $18\times 32 \times 32$ to ease the visualization of the resulting networks.

**Fig. 4:**
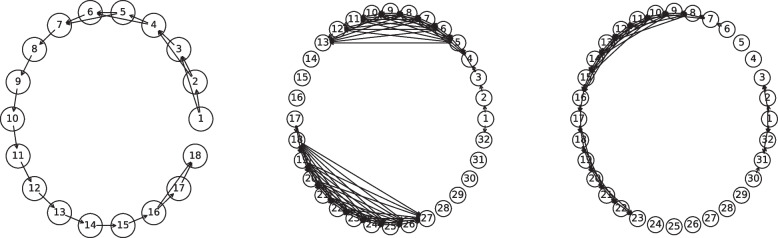
The resulting frame (left), row (center), and column (right) graphs learned by $\boxplus$-LGAM on COIL-20 data. Vertices are labeled according to their position in the dataset; vertex 7 in the row graph corresponds to the seventh row, for example. For the frame graph we used an L1 penalty of $9.2 \times 10^{-3}$; for the others, a penalty of $5 \times 10^{-3}$ was used; parameters were chosen manually

**Fig. 5:**
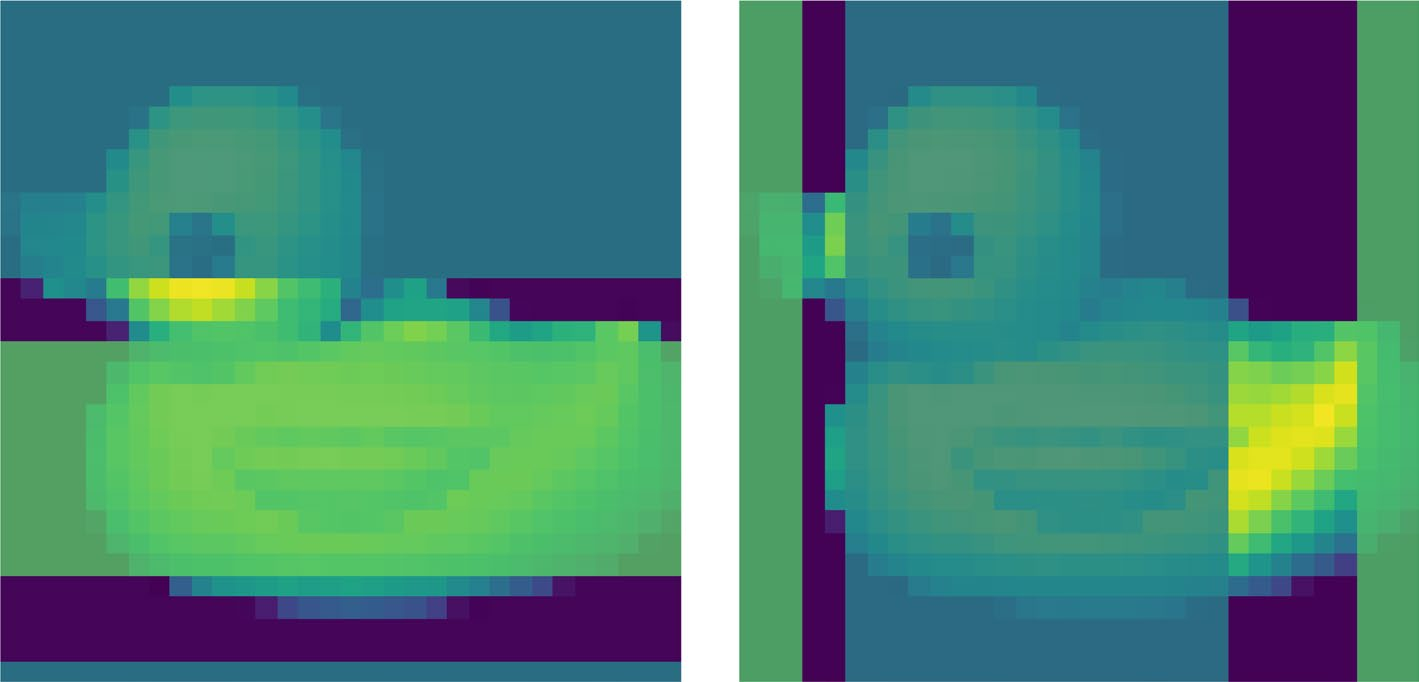
The row and column graphs learned by $\boxplus$-LGAM on COIL-20 data were each clearly divided into two separate components. These are highlighted here, with the row components in the left plot and the column components in the right plot

This example was chosen because it was the example used in the paper introducing the first Cartesian product conditional dependency methodology (BiGLasso [6]), and because it does not require any domain knowledge to understand the results. We applied $\boxplus$-LGAM to this video without preprocessing (beyond downsampling), using the same causal ordering as provided by the data. W display the resultant graphs in Fig. 4. We would expect the ground truth to be AR(1); each frame only depends on the previous. If we compare our learned frame graph to this, we achieve an MCC of 0.872. We can also see that the row graph naturally clusters into ‘duck head’ and ‘duck body’ clusters, and the column graph corresponds to ‘non-duck’ and ‘duck core’ clusters, implying that some structure has been learned for these axes as well (Fig. 5).

## Discussion

This paper addresses the problem of directed regulatory network inference. In particular, we have generalized the classical linear Gaussian additive noise model using a Kronecker-separable framework. Our method represents a first step in the construction of multi-axis gene regulatory inference models; there are many interesting future avenues of research. Below, we summarize the strengths and limitations of our method, and outline how future work could go about addressing these.

### Strengths

This paper brings multi-axis techniques into a DAG estimation framework. In doing so, we have shown how to replace the common independence assumption with the weaker Cartesian product assumption. We show how this improves performance over the analogous independence-assumption model, LGAM (in the Results section); this technique could be adapted to work for more advanced independence-assuming models as well (which we briefly discuss in the next section). By using the Cartesian product assumption, we gain the ability to learn cell and gene graphs simultaneously; this allows metrics for one graph to be used to learn the other graphs (in the Simulated scRNA-seq data section). On a theoretical level, our method achieves a standard statistical recovery rate; we have not lost anything asymptotically by removing the independence assumption (Theorem 1).

### Limitations and future work

Our method makes four assumptions about the data. Assumptions 1 and 2 (linear dependencies, Gaussian data) could be relaxed in future work by applying this Cartesian product framework to more complicated base distributions; this is conceptually straightforward (although the extent to which the theoretical results would carry over would require some investigation). Rather than assuming Gaussian noise, one could for instance assume Cauchy-noise ($\mathcal {C}$) for heavier tails:

$$\begin{aligned} \textbf{X} & = \textbf{C}_{\textrm{cells}}\textbf{X} + \textbf{X}\textbf{C}_{\textrm{genes}}^T + \textbf{E} \\ \textbf{E}_{ij} & \sim _{i.i.d.} \mathcal {C}\left( 0, 1\right) \end{aligned}$$

The Cartesian product assumption (Assumption 3) is central to our work. Depending on the scenario, it may not be suitable - for example, it does not take into account dependencies between genes in different cells, which can arise in real scenarios. One could change the model as follows to take these into account:

$$\begin{aligned} \textbf{X} & = \textbf{C}_{\textrm{cells}}\textbf{X} + \textbf{X}\textbf{C}_{\text {genes within cells}}^T + \textbf{C}_\textrm{cells}\textbf{X}\textbf{C}_{\text {genes between cells}}^T + \textbf{E} \\ \textbf{E}_{ij} & \sim _{i.i.d.} \mathcal {C}\left( 0, 1\right) \end{aligned}$$

The new $\textbf{C}_{\text {genes between cells}}$ term captures gene-gene dependencies that occur between cells connected in the $\textbf{C}_\textrm{cells}$ network. We considered a similar model for conditional dependency networks [9], but did not find much improvement; however, there is significant scope to improve the structure of such models to better match the biology of the scenario; the structure given above is not the only option. Such models would typically not be convex, even in the known-causal-ordering case. Determining the best structure would be a fruitful avenue for future work.

Finally, we assume that the causal ordering is known (Assumption 4). We make this assumption to ensure convexity of our objective. In principal, one could remove this restriction and fit our algorithm over the space of all DAGs; in doing so, we would loose the theoretical guarantees we provide and converge only to a local optimum. The variance-based heuristic (Theorem 4) may not be suitable for all types of data. However, Theorem 4 was created by lifting analogous results from the independence-assuming literature; our approach should be applicable to most heuristics in the literature as it did not depend much on the specifics of the heuristic.

Of particular interest for future work would be taking an approach similar to NOTEARS [28]. NOTEARS is a single-axis method which applies a regularizer that is 0 if and only if the input matrix is a DAG. This allows one to relax the problem to an easier domain (the space of all square matrices); it remains nonconvex, but can be continuously optimized. This approach is compatible with ours. Other advanced single-axis methods for gene regulatory network inference, such as GENIE3 [29] and PIDC [30], would require more thought to bring into a multi-axis framework.

Finally, our results (Theorems 2 and 3) put a cap on how lopsided data can be for our methods to work; this can be restrictive. Such results do not appear when one works with conditional dependency networks, which raises an interesting question for future work: are lopsidedness limitations fundamental to DAG-based models, or can multi-axis DAG models be created without such limitations?

## Data Availability

The code and seeds for datasets generated and/or analysed during the current studyare available in our GitHub repository, https://github.com/BaileyAndrew/Cartesian-LGAM, including that of Krumsiek et al. [22]. We implemented our algorithm in Python, using NumPy [31], on a personal computer (2020 M1 Mac with 8GB of RAM); the code for all experiments is available atthe same repository.

## Footnotes

1 An ‘oracle solution’ is the best possible parameter choice (measured by Kullback-Leiber divergence from the true distribution), and equals the true parameter choice when the model is not misspecified.

2 If $\alpha$ is close to 0 (i.e. the true distribution has heavier tails) or if the true distribution’s covariance matrix is ill-conditioned, then $\beta = \frac{4}{\alpha }$ is more likely.
